# Risk Categorization with Different Grades of Cervical Pre-Neoplastic Lesions - High Risk HPV Associations and Expression of p53 and RARβ

**DOI:** 10.31557/APJCP.2019.20.2.549

**Published:** 2019

**Authors:** D Ghosh, A K Roy, N Murmu, S Mandal, A Roy

**Affiliations:** 1 *Department of Pathology and Cancer Screening,*; 2 *Department of Signal Transduction and Biogenic Amines, *; 3 *Department of Epidemiology and Biostatistics, Chittaranjan National Cancer Institute,*; 4 *Institute of Medicine and Sagar Dutta Hospital, Kolkata, India.*

**Keywords:** Cervical cancer, histopathology, immunohistochemistry, papilloma viruses, tumour markers

## Abstract

**Objective:**

To identify high risk HPV associations by evaluating linked p16 overexpression and also the expression of p53 and RARβ together with histopathology for risk categorization of cervical pre-neoplastic lesions.

**Materials and Methods:**

Immunohistochemical staining was performed on 100 cases of cervical pre- neoplastic lesions for expression of biomarkers like p16, p53 and RARβ for comparison with haematoxylin/eosin (HE) findings. All the experimentally generated data were statistically analyzed.

**Results:**

In this study 70% cases showed overexpression of p16INK4A increasing progressively from CIN I to CIN II but reduced in CIN III (p <0.01). p53 oncoprotein expression was seen in 51% cases, again with increments from CIN I to CIN II with slight reduction in CIN III (p<0.01). Some 24% cases showed negative immunoreactivity for the putative tumor suppressor gene RARβ (p>0.05).

**Conclusion:**

Our study provides support for the idea that p16 can be used to identify associations with HPV , as well as having potential along with p53 and RARβ for categorizing cervical pre-neoplastic cases having a higher risk of neoplastic conversion. Thus it may be concluded that accurate risk categorization can be achieved with the help of genetic markers as well as histopathology.

## Introduction

Worldwide, cervical cancer is the fourth most common cancer and the second leading cause of death in women aged 15–44 years, with an estimated 569,847 new cases and 311,365 deaths in 2018. In India, cervical cancer is the second most common cancer in women with 96,922 new cases and 60,078 deaths in 2018 (Bray et al., 2018). Organised screening programmes are generally considered the milestone in cervical cancer prevention. 

Cervical cancer is preceded by pre-neoplastic lesions, cervical intraepithelial neoplasia (CIN) and histopathologically, it is classified as mild (CIN I), moderate (CIN II) and severe (CIN III). Approximately 1.5 per 1000 women in developed countries is diagnosed with CIN II/CIN III annually (Tainio et al., 2018). 

Human papilloma viruses (HPVs) are the main etiological factor in the occurrence of pre-neoplastic lesions and cervical cancer. High Risk-HPVs (HR-HPVs) are the main causal agent of cervical cancer and are present in 95% of cervical infections (Udar and Rader, 2014). In India, HR-HPV 16 and 18 are the most common HPVs and are responsible for 82.7% of invasive carcinoma (Mishra et al., 2016). The majority of HPV infections regress spontaneously without treatment, only small percentage of cases infected with persistent HR-HPV develop pre-neoplastic cervical lesions (Hwang et al., 2012) of which very few develop invasive cancer unless detected and treated (Udar and Rader, 2014; Guitrezz et al., 2015). In addition, it has been seen that many cases of CIN III remained stable for many years whereas patients with CIN I carried significant risk and progressed to malignancy within short period of time (Kim et al., 2001). This accentuates the importance of risk categorization diagnosis as well as identification of those pre-neoplastic lesions that are at highest risk of progression.

Histopathological examination is considered as the “gold standard” in the assessment of cervical lesions, however, it confers little or no information regarding the risk of persistence, progression or regression of cancer (Hwang et al., 2012; Wu et al., 2014). HPV infections and molecular events supersede cell cycle controls, the immune detection of cell proteins that are differentially expressed in infected cells is currently being considered for use as potential tumour and prognostic marker, to improve diagnostic accuracy as well as to identify those patients at risk for progression to cancer (Sarma et al., 2017). Under such perspective, biomarker study in combination with histopathology increases the sensitivity. 

HPV integration into the host genome is a critical step in the process of cervical carcinogenesis and cervical cancer which leads to increase in the expression of E6 and E7 viral oncoproteins that have the ability to inactivate p53 and retinoblastoma protein (pRb) respectively (Uyar and Rader, 2014). Inactivation of Rb protein leads to overexpression of p16INK4a thus it is considered as a surrogate marker for HR-HPV associated lesions and can discriminate integrated from non integrated HPV infection (Sarma et al., 2017; Lesnikova et al., 2009). p53 can be functionally inactivated in cervical carcinoma either by association with E6 or mutation in the gene (Stiasny et al., 2017). Due to these p53 abnormalities, cervical epithelial cell is unable to exit the cell cycle leading to genetic instability and are responsible for the development of cervical cancer (Godoy et al., 2014; Raju et al., 2015). Immunohistochemically, detection of wild type p53 is difficult due to very short half life but IHC can detect mutated p53 protein or oncoprotein thus rendering valuable prognostic information and can be useful for risk categorization. 

RARβ (Retinoic Acid Receptor Beta) is a putative tumour suppressor gene and a member of nuclear receptor RAR (Retinoic Acid Receptor). It is a negative regulator of viral oncogenes E6 and E7 thus decreased expression of RARβ may be an important step towards malignant progression of HPV-positive cells (Ivanova et al., 2002, Wongwarankana et al., 2018). Therefore it can be used as a useful biomarker to identify the cases that are at higher risk of cancer conversion and is a striking feature in human carcinomas including head and neck, breast, oral, pancreas and carcinoma of uterine cervix (Geisen et al., 2000). 

In the present study, we aimed to evaluate high grade HPV association by p16, expression of p53 and RARβ in pre-neoplastic cervical lesions for risk categorization.

## Materials and Methods

In this study a total of 100 cervical pre-neoplastic cases including 9 associated lesions were selected from patients attending CDC OPD (Cancer Detection Centre Out Patient Department) of Department of Pathology and Cancer Screening at Chittaranjan National Cancer Institute, Kolkata, during the period of 2014 to 2017 and punch biopsy was collected for further analysis at the Department of Pathology and Cancer Screening. Written informed consent was obtained from all patients and the study was approved by Institutional Ethical Committee. Biopsy specimen was fixed in 10% formalin and processed within 24 hours. After the routine processing, paraffin tissue blocks were made and cut on a microtome in serial sections and deparaffinised sections were then stained with haematoxylin and eosin (HE). The slides were reviewed and graded according to the criteria of the World Health Organisation as CIN I, CIN II, CIN III and other associated cervical lesions (Harsh, 2010) by distinguished pathologist.

*Immunohistochemistry*

For immunohistochemistry of p16, p53 and RARβ protein expression, IHC was performed on deparaffinised sections and positive controls according to the protocol of IHC World (http://www.ihcworld.com/protocol_database.html) with slight modification and commercially available kit (IHC Select- HRP/DAB Kit Millipore) was used for detection. Briefly, antigen retrieval was carried out by heating the slides in 0.01 M citrate buffer, pH 6.0 in microwave oven for 10 minutes. Endogenous peroxidase was blocked by 3% hydrogen peroxide in water for 10 minutes. Non specific binding was blocked by blocking reagent for 5 minutes and slides were not washed down. Monoclonal antibodies against p16 (clone 2D9A12; 1:600; abcam), p53 (clone BP53-12; 1:400; Sigma), and RARβ (clone EPR2017, 1:50; abcam) were applied and incubated for 20 minutes at 25^o^C for p16 and overnight at 4^o^C for p53 and RARβ . Further, sections were sequentially incubated with secondary antibody for 10 minutes. Next Streptavidin HRP (Horseradish Peroxidase) was applied and incubated for 10 minutes. Chromogen DAB (3,3’- Diaminobenzidine) solution was freshly prepared and added to the tissue sections and incubated for 10 minutes and then counterstained by Meyer’s haematoxylin for 1 minute. The slides were then passed through a series of graded alcohol and xylene and mounted with DPX. Negative control sections were processed by eliminating the use of respective primary antibodies. After each step slides were washed with rinse buffer thoroughly and only excess liquid around the section were blotted with tissue paper. 

*Scoring method*

Scoring of p16 immunohistochemistry was done according to Chin Ping Han et al., (2009). In each case a total of 1,000 cells were counted at 40X and was scored according to the intensity of the nuclear or cytoplasmic staining (no staining-0; weak staining-1; moderate staining-2; strong staining-3) and the extent of stained cells (0%-0; 1–10%-1; 11–50%-2; 51–80%-3; 81– 100%-4). The final score was determined by multiplying the intensity and extent of positive cells ranging from 0 to 12. Score of 4 to 12 is denoted as positive or overexpression and 0 to 3 score is denoted as negative. 

**Figure 1:**
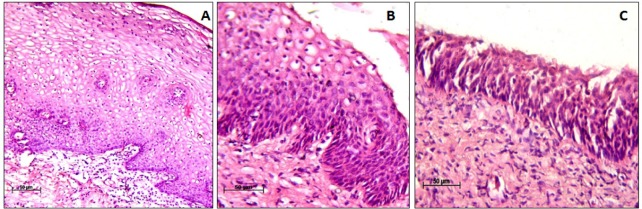
Haematoxylin/Eosin Staining. (A), Cervical Intraepithelial Neoplasia (CIN) I x 10X; (B), CIN II x 20X; (C), CIN III x 20X

**Figure 2:**
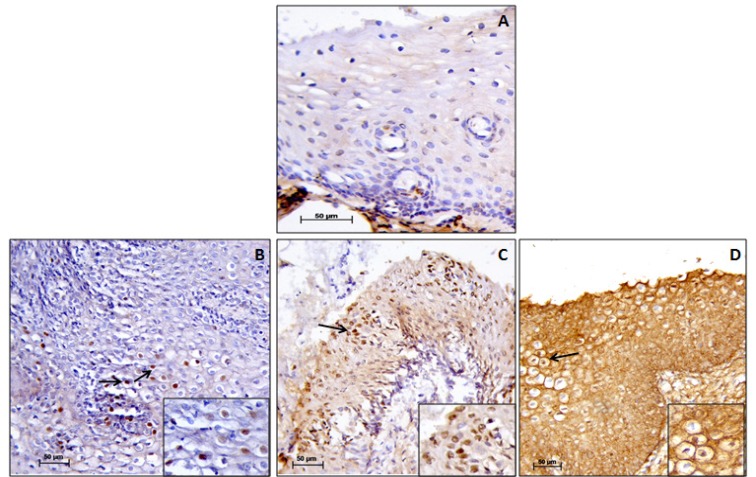
Immunohistochemistry of p16. (A), p16 immunonegativity (score 0); (B), rare singly dispersed p16 staining (score 1); (C), patchy but strong p16 staining (score 3); (D), strong and diffused p16 staining (score 4). The magnification of the main and insert image is 20X and 40X, respectively. Scoring according to the intensity of the staining. Black arrows denote p16 immunopositive cells

**Figure 3:**
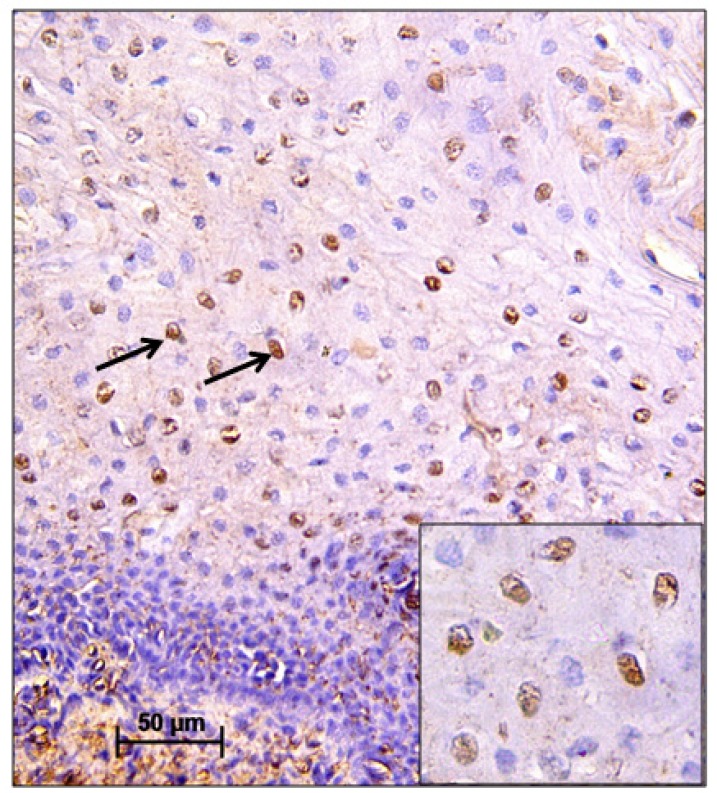
CIN I Showing Nuclear Immunopositivity for p53 (Black Arrows). The magnification of the main and insert image is 20X and 40X, respectively

**Figure 4:**
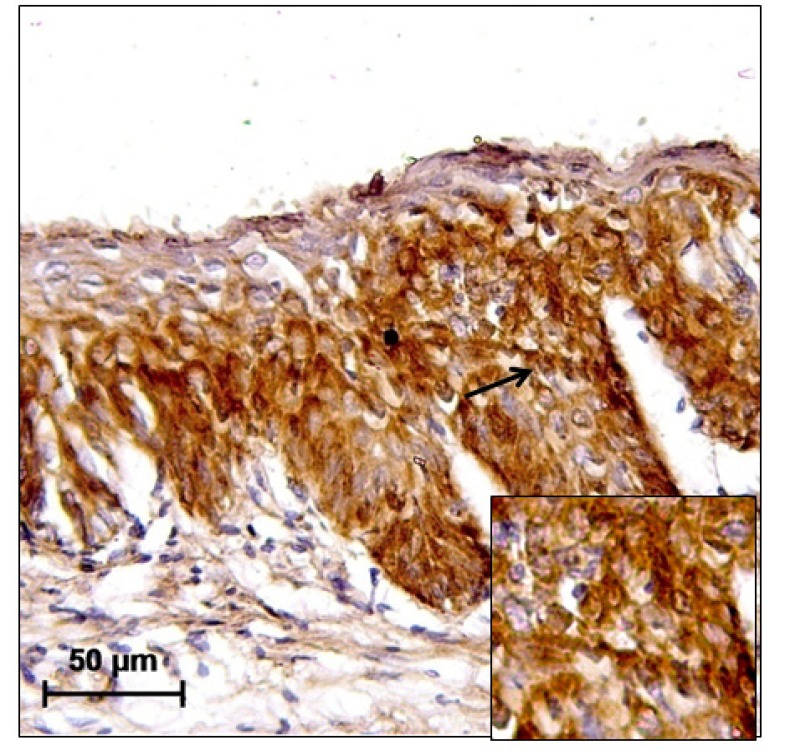
CIN II Showing Intense Diffuse RARβ Immunoreactivity in Both Nuclei and Cytoplasm (Black Arrow). The magnification of the main and insert image is 20X and 40X, respectively

**Figure 5:**
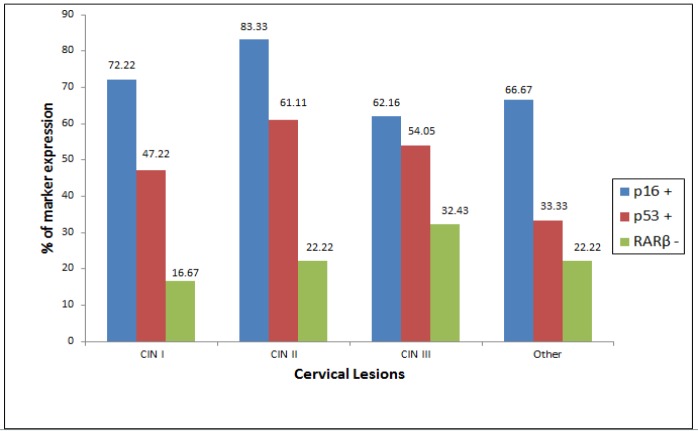
p16 Overexpression, p53 Immunopositivity and RARβ Immunonegativity with Different Grades of Cervical Lesions

**Table 1:** Immunohistochemical Expression of p16, p53 and RARβ in Pre-Neoplastic and Other Associated Lesions of Uterine Cervix

Parameters	Biomarkers	No of cases (Total cases = 100 of age range 20-55 yrs)	CIN I n=36	CIN II n=18	CIN III n=37	Other Associated lesions n=9	p values
Expression of Biomarkers	p16 +	70 (70%)	26 (72.22%)	15 (83.33%)	23 (62.16%)	6 (66.67%)	<0.01*
	p53 +	51 (51%)	17 (47.22%)	11 (61.11%)	20 (54.05%)	3 (33.33%)	<0.01*
	RARβ -	24 (24%)	6 (16.67%)	4 (22.22%)	12 9 (32.43%)	2 (22.22%)	>0.05 (NS)
					(p<0.001)**		

Percentage represents within the different grades of cervical lesions; *Statistically Significant; NS- Not Significant; **Statistically significant within the group

The p53 and RARβ expression were analyzed semi quantitatively by counting 1,000 cells at 40X under light microscope. p53 immunoreactivity was considered positive in cases having more than 10% positive nucleus (Qin et al., 2002). In case of RARβ, cells having no or less than 10% nuclear and cytoplasm positive cells were considered negative and included in the study (Chakravarti et al., 2003). 

*Statistical Analysis*

Statistical Analysis was performed with the help of Epi Info (TM) 7.2.2.2. EPI INFO is a trademark of the Centers for Disease Control and Prevention (CDC). Descriptive statistical analyses were performed to calculate the means with corresponding standard deviations (s.d.). Test of proportion was used to find the Standard Normal Deviate (Z) to compare the difference proportions and Chi-square (ϰ^2^) test was performed to find the associations. In the cases where one of the cell frequencies were less than 5 corrected Chi-square (ϰ^2^) was used to find the association between variables. t-test was used to compare the means. p<0.05 was taken to be statistically significant.

## Results

100 cases of cervical pre-neoplastic lesions including 9 other associated cervical lesions were included in the study. The mean age of the patients was 42.21 ± 9.08 years with range 20 – 55 years and the median age of the patients was 42 years. 

Histopathologically, there were 36 cases (36%) of CIN I, 18 cases (18%) were CIN II, 37 cases (37%) were CIN III (Figure 1) and 9 cases (9%) with other pathological conditions including chronic cervicitis (2 cases), koilocytotic changes (4 cases), reactive cellular changes (RCC) (1 case) and condyloma (2 cases). 

Overexpression of p16 was seen in 70% (70/100) of cases and test of proportion showed that overexpression was significantly higher in CIN I and CIN II (CIN I – 72.22%, CIN II – 83.33%) but decreased in CIN III (62.16%) (Figure 2). 66.67% cases of 9 other associated lesions 6 cases (condylomas 2, cervicitis 1, RCC 1, Koilocytosis 2) showed positivity for p16. Test of proportion showed that proportion of expression was significantly higher in all the four layers of the cervical tissue (68.57%) followed by expression in superficial and intermediate layer (15.71%) (p<0.001). p53 positivity was seen in 51% (51/100) cases, which increased from CIN I to CIN II (CIN I – 47.22%, CIN II – 61.11%) and slightly decreased in CIN III (54.05%) (Figure 3). 33.33% other associated lesions showed p53 positivity (condylomas 1, cervicitis 1, RCC 1). In case of RARβ, 24 cases (24%) showed negative immunoreactivity and remaining 76 cases (76%) showed immunopositivity (Figure 4). The test of proportion showed that RARβ negativity was significantly higher in CIN III (32.43%) than CIN II (22.22%) and CIN I (16.67%) (p<0.001). 2 cases (22.22%) with other pathological condition showed immunonegativity for RARβ. 

p16 showed significant overexpression followed by p53 which showed moderate expression in all the grades of cervical pre-neoplastic and associated lesions (p<0.01) whereas RARβ immunonegativity was statistically not significant (p>0.05) (Table 1) (Figure 5).

There were 6 cases (6%) out of 100 that showed overexpression of p16, immunoreactivity for p53 and negative staining for RARβ of which 1 case (16.66%) was CIN I, 1 case (16.67%) were CIN II, 3 cases (50%) were CIN III and 1 case (16.67%) was of other associated cervical conditions.

## Discussion

Cervical cancer is the leading cause of mortality and morbidity among women in the developing countries than developed countries. Association of high risk HPVs are considered as the most important etiological factor linked to cervical neoplastic and pre-neoplastic lesions (Uyar and Rader, 2014). Routine histology is the gold standard for pathological characterization of cases for risk assessment but it has certain limitations such as many cases of cervical cancer skip the pre-invasive cervical lesions before developing malignancy (Lesnikova et al., 2009) hence biomarker study along with histopathology can be accurate in predicting the outcome of the individual pre-neoplastic cases. 

p16 overexpression has been considered as sign of integration of HPV (Chuerduangphui et al., 2018; Izadi Mood et al., 2012). IHC expression of p16 was observed only in dysplastic or neoplastic cells and was never expressed in normal cervical epithelium (Lesnikova et al., 2009) and according to many authors p16 expression appears to be a robust, specific sensitive biomarker of cervical neoplasia (Dray et al., 2005; Chuerduangphui et al., 2018). In our study p16 overexpression was highly significant and showed positive immunoreactivity in majority of the cervical pre-neoplastic lesions. Overexpression progressively increased from CIN I to CIN II but reduced in CIN III. Similar results were observed in different studies (Klaes et al., 2001; W Feng et al., 2007; Izadi Mood et al., 2012; Sarma et al., 2017) in which they also reported that there is a decreased p16 overexpression in invasive cervical cancer in comparison to high grade cervical pre-neoplastic lesions. It has been seen that overall p16 immunoexpression in cervical pre-neoplastic lesions, described as overexpression, ranges from 31 to 100% (Tsoumpou et al., 2009; Von Knebel et al., 2012; Wu et al., 2014). A study by Lesnikova et al., (2009) has seen that most cases of CIN I and large proportion of CIN II and CIN III can be expected to regress spontaneously. The rate of regression of CIN III is almost threefold in comparison to the progression of invasive carcinoma. Thus, the decreased expression of p16 in high grade cervical lesions justifies the theory of regression. On the other hand many research articles expressed view that the p16 expression increases progressively with change of histopathological grade such as CIN I has the lowest expression and CIN III has the highest (Tsoumpou et al., 2009; Wu et al., 2014; Von Knebel et al., 2012) which subsequently progress to carcinoma cervix.

In the present study it was also found that 6 out of 9 cases of other associated lesions showed overexpression for p16, whereas many cases with histopathologically high grade lesion showed p16 negativity. Therefore, it appears that apart from p16 positivity of pre-neoplastic lesions, other cases such as condylomas also express p16 implying that HPV infectivity found in any of histological pattern barring CINs, are important and again shows the limitation of histopathology alone for risk prediction of cervical lesions. 

p53, tumour suppressor gene, plays an important role in protection against development of cancer. However, mutation or conformational changes from suppressor to mutant p53 results in p53 oncoprotein expression (Raju et al., 2015). In our study, 51% of the cases showed positivity for p53 in cervical pre-neoplastic lesions and was significantly linked to histopathological grades CIN I to CIN II and its expression reduced in CIN III. A study by Grace et al., (2003) demonstrated that there is expression of p53 protein in the early stages of cervical lesions like CIN I and CIN II whereas some studies have reported that the p53 expression is a late event and was seen in advanced cervical intraepithelial lesion (CIN III) and invasive cancer (Goel et al., 2012). An Indian study by Raju et al., (2015) showed that there is high expression of p53 from LSIL to HSIL but another Indian study by Shukla et al., (2014) found that low percentage of p53 expression in CIN and a moderate expression of p53 in cervical carcinoma whereas an International study by W Feng et al., (2007) reported that p53 expressed in both cervical cancer and cervical dysplasia. Though there are some controversies in relation to p53 expression and histopathological grading, from the above studies it can be said that immunodetection of p53 oncoprotein in any cervical lesions, irrespective of its histopathological grade, carries significant risk of conversion and thus can be used as an important biomarker. 

RARβ exerts an inhibitory effect on expression of viral oncoproteins E6 and E7 thus decrease in RARβ leads to the development of pre-neoplastic cervical lesions and cancer (De-Castro Arce et al., 2007). In this study, the expression of RARβ decreased from CIN I to CIN III. Negative immunoreactivity for RARβ was seen in 24% cases of CINs and was most common in high grade lesions (CIN III) followed by CIN II and CIN I. A study by Wongwarangkana et al. (2018) also expressed similar view and found that decrease expression of retinoic acid receptor occurs early in the development of cervical carcinoma and has been linked to CINs. Narayan et al., (2003) found that in the LSIL group, 11% had low RARβ expression whereas, in the HSIL group, 60% had a complete lack of RARβ expression. Another study by Choi et al., (2007) discovered that all normal tissues highly express RARβ protein, whereas no staining was detected in 43% of the squamous cell carcinoma. According to Ivanova et al., (2002), 40% decrease of RARβ2 mRNA was found in cervical squamous cell carcinoma. 

In this study it has been found that out of 100 cases there are 6 cases showing p16 overexpression along with p53 positivity and RARβ negativity. These cases along with CIN III followed by CIN II and CIN I respectively have the highest probability of malignant transformation. In the International arena similar results have been observed by Yim et al., (2005), Hwang et al., (2012), Lesnikova et al., (2009), Uyar and Rader (2014).

We also found that out 49 p53 negative cases, 31 cases were p16 positive and 11 cases were RARβ negative, of which 10 were CIN III. Barring p53 positivity it has been reported that in high risk HPV-positive cases decrease expression of RARβ may be an important step on the way towards malignant progression (De-Castro Arce et al 2007; Ivanova et al., 2002).Thus it is expected that high grade cases that are p53 negative but p16 positive and RARβ negative also carry significant risk for malignant transformation.

Therefore, from the study it can be concluded that p16, p53 and RARβ are equally important individually for risk categorization along with histopathological grading. However, it is pertinent to say that the combined marker study of these three markers including histopathology can be used for more accurate risk categorization and probably carries highest importance or value in categorizing risk and predicting cancer progression in cervical pre-neoplastic lesions.

In conclusion, we conclude that histopathological evaluation alone is inconclusive for predicting risk in cervical lesions. The use of biomarkers such as p16, p53, RARβ in conjunction with histopathology could greatly improve the accuracy, precision and sensitivity of cervical screening program for risk categorization of pre-neoplastic and other lesions of cervix. These markers thus may be helpful particularly in developing countries for early risk assessment where the genetic testing of pre-neoplastic lesions as well as cancer treatment is too expensive and out of reach of most of the people in India. 

## Limitation of the study

Statistically the accuracy level of the study would be higher if more number of patients could be included in the study. Secondly, for further enhancement of the significance of the study, the cases needed to be followed up at a regular intervals for a longer period of time as it is one of the important steps to identify the outcome of these cases which could strongly support the predicted result and would have justified the conclusive role of these markers for risk categorization.

## Funding Statement

This research received no specific grant from any funding agency in the public, commercial or not-for-profit sectors.
